# Do hedonic- versus nutrition-based attitudes toward food predict food choices? a cross-sectional study of 6- to 11-year-olds

**DOI:** 10.1186/s12966-017-0618-4

**Published:** 2017-11-25

**Authors:** Lucile Marty, Maud Miguet, Marie Bournez, Sophie Nicklaus, Stéphanie Chambaron, Sandrine Monnery-Patris

**Affiliations:** 1 Centre des Sciences du Goût et de l’Alimentation, AgroSup Dijon, CNRS, INRA, Univ. Bourgogne Franche-Comté, F-21000 Dijon, France; 2 University Hospital of Dijon, Pediatric Unit, Dijon, France; 3 Centre des Sciences du Goût et de l’Alimentation CSGA INRA, 17 rue Sully, BP86510, 21065 Dijon Cedex, France

**Keywords:** Attitudes, Food choices, Children, Nutrition, Hedonic

## Abstract

**Background:**

Implicit and explicit attitudes are potential precursors of food choices and combine affective and cognitive components that can vary in their relative dominance. Yet, the affective and cognitive components of attitudes toward food can lead to distinct predisposition toward a food item and potentially to different food choices. In the food domain, the affective component pertains to the hedonic tone of consumption, while the cognitive component encompasses nutritional value or health consequences of food. The present study investigated whether hedonic- versus nutrition-based implicit and/or explicit attitudes toward food predicts children’s healthy versus unhealthy food choices.

**Methods:**

A total of 63 children (age range = 6.3–11.5) participated in a 90-min session at 5 pm (i.e., afterschool snack time in France). The children were asked to choose five food items from a buffet featuring five healthy and five unhealthy sweet foods pretested as being highly liked. Children ate what they had chosen. Moreover, their implicit attitudes were assessed with a pairing task in which children were presented with 10 food triplets and asked to choose two food items that “best go together”. For each triplet, foods could be paired according to their hedonic or nutritional characteristics. Explicit attitudes were assessed with a task in which children placed each of 48 food items into one of the following categories: “yummy”, “yucky” (i.e., hedonic categories), “makes you strong”, or “makes you fat” (i.e., nutritional categories).

**Results:**

Both implicit and explicit attitudes significantly influenced children’s food choices. We observed that children with more hedonic-based implicit or explicit attitudes toward food were more likely to choose healthy food options from the buffet. Conversely, children with both implicit and explicit nutrition-based attitudes chose less healthy foods.

**Conclusions:**

Hedonic-based attitudes toward food seem to drive healthier food choices in children compared with nutrition-based attitudes in this particular eating context. These findings suggest that pleasure from eating might be an ally with regard to healthy eating among children. Additional research is needed to understand the etiology of children’s attitudes toward food in order to provide insights on how to shape adequate children’s attitudes to guide them toward healthy food choices.

**Electronic supplementary material:**

The online version of this article (10.1186/s12966-017-0618-4) contains supplementary material, which is available to authorized users.

## Background

Pleasure from eating is an innate driver of food consumption, and food likes and dislikes are strong predictors of children’s food choices [1, 2]. However, children’s food preferences generally do not align with dietary recommendations: they typically rate energy-dense foods as the most liked and vegetables as the least liked foods [3–7]. To encourage children to adopt a healthy diet, parents, caregivers, and national campaigns display nutritional information [8]. In this context, children acquire an early awareness about both the hedonic and nutritional values of food. Five-year-olds are able to classify food products considering both hedonic and nutritional perceptions [9]. Interestingly, qualitative studies have shown that children consider both hedonic and nutritional perceptions as factors that influence their food choices [10, 11]. A better understanding of the factors that underlie healthy or unhealthy eating in children is of particular importance because eating habits remain stable from childhood to adulthood [12, 13]. However, surprisingly sparse research has examined the relative influence of hedonic versus nutritional considerations on children’s food choices [14]. To fill this gap, the current study investigated the relationship between children’s attitudes toward food (in particular, the relative dominance of nutrition- versus hedonic-based attitudes) and children’s choices of healthy versus unhealthy foods.

Attitudes are commonly viewed as memory associations between objects and their evaluations that guide behavior toward these objects [15, 16]. Attitudes combine cognitive and affective components that can vary in their relative dominance to form global evaluations of an object [17–20]. In the food domain, the affective component pertains to the sensations, feelings and emotions experienced in response to a food item (e.g., the hedonic tone of consumption), whereas the cognitive component encompasses the positive and negative attributes and beliefs about a food item (e.g., its nutritional value and health consequences) [21]. Importantly, Dubé and Cantin showed that the relative dominance of affective or cognitive components of attitudes toward a food item influences eating behavior [22]. In fact, the affective and cognitive basis of attitudes toward food can lead to distinct evaluative consequences. The affective component of attitudes toward a food item (e.g., chips) can lead to a positive evaluation because it is tasty, whereas the cognitive component of the attitude can lead to a negative evaluation because it is unhealthy. One’s global evaluation of chips may differ depending on whether one favors the affective or cognitive component. Thus, primarily hedonic-based (i.e., affect-based) or nutrition-based (i.e., cognitive-based) attitudes toward food can lead to different food choices.

In addition, numerous psychology studies have demonstrated the influence of non-conscious processes on decision making [23–25], and the distinction between implicit and explicit attitudes has emerged [26]. Implicit attitudes likely operate automatically in a non-conscious fashion, whereas explicit attitudes are likely retrieved deliberatively under conscious control [26]. Several attempts have been made to develop predictive models combining implicit and explicit attitudes and their influences on behavior [27–29]. Three major models have been proposed in the literature and tested in a food context by Perugini [30]: (1) additive (the two types of attitudes explain different proportions of the variance of behavior), (2) double dissociation (implicit attitudes predict spontaneous behaviors, whereas explicit attitudes predict deliberative behaviors), and (3) multiplicative (implicit and explicit attitudes interact to influence behavior). Perugini observed that the double dissociation model of Wilson et al. [27] predicted eating behaviors: implicit attitudes were significantly related to spontaneous eating behaviors (namely, the choice of a snack or fruit at the end of the experiment), whereas a significant relationship was found between explicit attitudes and deliberative behavior (namely, the self-reported consumption of snacks and fruit during an average week) [30]. Interestingly, König et al. recently showed that both explicit and implicit attitudes were independent precursors of food choice in a binary-option choice task. Conversely, in a multiple-option choice task, the effects of explicit and implicit attitudes were qualified by an interaction. The participants served themselves more sweets than fruit when their implicit and explicit attitudes revealed a consistent preference for sweets, as well as when one attitude type (i.e., either the implicit or the explicit one) indicated a preference for sweets while the other indicated a preference for fruit [31]. Contrary to Perugini’s conclusion [30], these results support the additive model using a binary-option food-choice task and suggest a shift from the additive to the multiplicative predictive model when multiple food options are offered. In summary, although both implicit and explicit attitudes influence eating behaviors, how they interact to predict eating behavior remains unclear.

Only few studies have investigated both implicit and explicit attitudes toward food in children [32–34] and, to our knowledge, the relationships between implicit and explicit attitudes and food choices has never been investigated in children. In a recent study, a new method was developed to assess hedonic versus nutritional basis of implicit and explicit food-related attitudes in children [34]. This new method included two tasks appropriate for use with children from 5 to 11 years old. The implicit attitudes were assessed with a pairing task, in which children were presented with food pictures triplets and asked to choose two food items that “best go together”. For each triplet, foods could be paired according to their hedonic or nutritional characteristics. Explicit attitudes were assessed with a categorization task in which children placed food items into one of the following categories: “yummy”, “yucky” (i.e., hedonic categories), “makes you strong”, or “makes you fat” (i.e., nutritional categories). For both tasks, for each trial, children had to choose between a hedonic-based and a nutrition-based answer. It was assumed that the proportion of hedonic- or nutrition-based answers would reflect the relative dominance of the hedonic or nutritional basis of implicit and explicit attitudes. The first study using these two tasks revealed an increase in implicit hedonic-based attitudes but a decrease in explicit hedonic-based attitudes with school level [34]. The tasks were also used to compare attitudes towards food between children with or without overweigh [33]. The results showed no difference in children’s responses in the implicit task based on their weight status, but children who were overweight were more likely to make nutrition-based categorizations than children who were normal-weight.

Following on from these results, the present study investigated whether the relative dominance of the nutritional (i.e., cognitive) versus hedonic (i.e., affective) components of implicit and explicit attitudes toward food are associated with children’s choices of healthy versus unhealthy foods. During an experimental session, children chose five of 10 highly appreciated healthy or unhealthy sweet food items to compose a snack they had to eat. Moreover, children’s attitudes toward food were assessed using the implicit pairing task and the explicit categorization task developed by Monnery-Patris et al. [34]. Based on the literature regarding adults [30, 31], we assumed that both implicit and explicit attitudes would predict the healthiness of children’s food choices. On one hand, nutritional interventions commonly assume that knowledge about the nutritional value of food improves the nutritional quality of children’s diets [35–37]. Based on this perspective, having nutrition-based attitudes toward food should drive healthier food choices in children. On the other hand, cross-cultural studies of adults have highlighted the positive relationship between hedonic perceptions of food consumption and overall health status [38, 39]. In addition, some evidence suggests that emphasizing hedonic considerations toward food may drive healthy eating behaviors in children [40]. Thus, it could be assumed that hedonic-based attitudes toward food would drive healthier food choices in children.

## Methods

### Participants

A power calculation to detect a large effect (*f* ^*2*^ = 0.35), assuming three predictors in a linear multiple regression, led to a sample size of 35 participants for 80% power at *α* = 0.05. The expected effect size was based on the results of König et al. (2016) who investigated the influence of implicit and explicit attitudes on food choices in a multiple-option food choice task [31]. A total of 63 children participated in this study (mean ± SD age = 8.99 ± 1.51, age range = 6.3–11.5 years, mean ± SD BMI (Body Mass Index) z-score = 1.65 ± 1.92, BMI z-score range = −1.74–5.74; 31 girls, 32 boys). Children were recruited from 6 to 11 years old because the implicit pairing task and the explicit categorization task were developed and adapted for this age range. Moreover, based on the results of a previous study using the same method [33], children varying in weight status were recruited to maximize the attitudinal variability of the sample. Children were recruited from a population registered in the Chemosens Platform’s PanelSens database. This database was declared to the relevant authority (Commission Nationale Informatique et Libertés; CNIL; n°1,148,039). Children with high body mass index z-scores (z-BMI) were specifically recruited from pediatric weight care consultations at a local hospital. The study was conducted in accordance with the Declaration of Helsinki and approved by the local ethical committee (Comité pour la Protection des Personnes EST-1 Burgundy, file number: 2015-A01547–42). Based on a recruitment questionnaire, children with food allergies were excluded. Written informed consent was obtained from both parents before their child’s participation. We certify that all applicable institutional and governmental regulations concerning the ethical use of human volunteers were adhered to throughout this study.

### Overview

The children and their parents were invited to a 90-min session after school (i.e., from 5 to 6:30 pm during weekdays) that occurred in our laboratory. The experiment was conducted with a maximum of 8 children per session and a minimum of 3. Children in the same session did not know each other. Parents were asked not to give their children a snack during the afternoon before the experiment. We welcomed the children and their parents in a waiting room, and only the children were invited to enter the experimental room. First, the children were asked to choose five snacks from an individual buffet and had time to eat what they had chosen. They made their choices individually but consumed their five snacks together at the same table. This protocol (i.e., first, individual choices, then commensal consumption) has been selected since it is very close to the meal proceedings at schools in France, in particular for afternoon snack. Hidden video cameras recorded the food choices of the children without their knowledge. Each individual buffet was separated from the others by two partitions to avoid interactions between children and to limit peer influence. Then, we measured the children’s attitudes toward food by placing them at separated tables. They performed an implicit and an explicit task on a computer. The tasks were self-administered, but experimenters were present to provide the initial instructions and were available for consultation if needed. Next, children individually rated their liking and healthiness perception of the foods offered in the buffet. Finally, the children’s height and weight were measured. Before leaving, the children received a certificate of participation, and their parents received a €20 voucher.

### Evaluation of food choices

Ten sweet foods, including 5 healthy foods (red apple, banana, kiwi, applesauce and strawberries) and 5 unhealthy foods (donut, chocolate cake, Smarties®, Kinder Bueno® and Gold Bears®) were served to the children in individual buffets (Table 1). These foods were selected based on a pre-testing of children’s familiarity with these foods conducted with a separate group of 61 children recruited from children’s holiday centers. During face-to-face interviews, the children were presented with pictures of the foods from the Food4Health pictures base [41] and reported whether they had ever tried them.

**Table 1:** Familiarity, energy density (ED) and portion size of the healthy and unhealthy foods served in the buffet

	Children who had tried the food (%) (pretest: *n* = 61)	ED (kcal/100 g)	Portion weight (g)	Portion energy (kcal)
Red apple	100	53.2	30 ± 1	16 ± 0.5
Banana	100	93.6	30 ± 1	28 ± 0.9
Kiwi	93	57.7	30 ± 1	17 ± 0.6
Applesauce	100	54.0	40 ± 1	22 ± 0.5
Strawberries	100	28.5	30 ± 1	9 ± 0.3
Donuts	90	400	22 ± 1	88 ± 4.0
Chocolate cake	100	430	15 ± 1	65 ± 4.3
Smarties®	93	464	15 ± 1	70 ± 4.6
Kinder Bueno®	100	572	11 ± 1	63 ± 5.7
Gold Bears®	100	345	21 ± 1	72 ± 3.5

Values are shown as the means ± SD. Energy density was obtained from the French food composition database [42] or from the food packaging when available

**Fig. 1:**
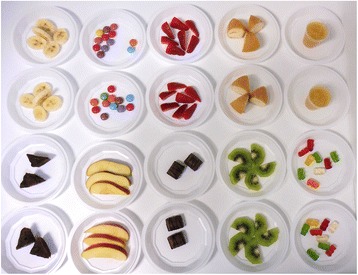
Picture of an individual buffet

An individual buffet with 20 small plates (two plates of each food) was prepared for each child in the study (Fig. 1). The foods were not presented in packets. The position of the healthy and unhealthy foods was counterbalanced: two healthy foods at the front and three at the back of the table, alternatively placed, versus three unhealthy foods at the front and two at the back of the table, alternatively placed. Under this predetermined position, healthy and unhealthy foods were randomly presented. Hidden video cameras were placed above each buffet to record the food choices of the children without their knowledge.

The video camera began recording before the children entered the experimental room. When the children entered, they were given a tray and assigned to their individual buffet. The children chose five plates to compose their snack. The children were told: “Today, we start the session by a snack! Each of you has an individual buffet prepared. You can choose five food items from your buffet and keep them on your tray. When you have made your choices, you can sit at the table with the other children to eat.” The children were able to choose two plates of the same food. They chose foods alone among the foods displayed in their individual buffet. Then, they ate together around the same table on which a glass of tap water (200 mL) and a napkin were available for each of them. They were told that they could leave some food on the plates if they did not want to eat anymore. When they finished, they left their tray on a trolley. After the participants left, the videos were watched. The number of healthy foods chosen by each participant was counted, and the time spent choosing the five food items was recorded.

### Characterization of children’s attitudes toward food

#### Implicit pairing task

Eleven triplets of food item pictures were successively presented, including an initial training triplet (strawberries, raspberries and whipped cream) followed by 10 test triplets (chicken, steak and French fries; baguette, butter and Nutella; tomatoes, olive oil and mayonnaise; cigarette cookies, chocolate chip cookies and applesauce; white bread, baguette and jam; waffle, pancakes and jam; plain cookies, chocolate chip cookies and applesauce; chicken, French fries and potato wedges; fruit salad, cigarette cookies and applesauce; and pasta, rice and steak) presented in a random order [34]. For each triplet of foods, children were asked to “choose the two foods that best go together”. The children were able to perform hedonic associations by pairing two food items that are typically consumed together (e.g., steak with fries or chicken with fries) or a nutritional association by pairing the two nutritionally similar food items (e.g., steak with chicken because they both belong to the meat category). The instructions and names of each food item were read aloud by the tablet computer when the food pictures appeared on the screen. For each triplet, the children answered by touching the two pictures on the screen that they thought matched. This action was recorded by the program, and no feedback was given. The pairing task required approximately 5 min. An implicit hedonic score based on the percentage of hedonic associations was calculated for each child (range: 0–100%).

#### Explicit forced-choice categorization task

A total of 51 food pictures were successively presented on a touch-screen tablet, including 3 for initial training (banana, lollipop, and rice) followed by 48 randomized test food item pictures: 9 fruits (red apple, grapefruit, orange, melon, grapes, pear, kiwi, nectarine, and fruit salad), 10 vegetables (tomatoes, carrots, cucumber, string beans, peas, vegetable soup, zucchini, mixed vegetables, ratatouille and lentils), 6 animal proteins (fish fillet, salmon steak, beef steak, lamb chop, fried egg, and omelet), 4 cheeses (Gruyere, Camembert, Roquefort, and goat cheese), 4 high-fat dishes (hamburger, lasagna, pizza, and French fries), 3 salted snacks (chips, pretzels, and small quiche), 5 sweet snacks (croissant, plain cookies, candies, chocolate, and candy bar), and 7 desserts (chocolate éclair, strawberry ice-lolly, crème caramel, ice cream, apple pie, chocolate cake, and chocolate pudding). The children chose whether each food item was “yummy” or “yucky” (poles of the hedonic dimension) or whether it “makes you strong” or “makes you fat” (poles of the nutritional dimension) by touching the corresponding pictogram on the tablet. Another category, “I don’t know this food”, was also available. The meaning of each pictogram was provided for the 3 first food items. The tablet computer read aloud the instructions and the name of each food item. Their choice was recorded by the program, and no feedback was provided. This task lasted approximately 10–15 min. An explicit hedonic score based on the percentage of hedonic answers (i.e., “yummy” or “yucky”) for known foods was calculated for each child (range: 0–100%).

#### Validation of the implicit pairing task and the explicit forced-choice categorization task

To confirm the validity of these measures, a pre-test with 27 different children (mean age = 8.45 years) was initially performed to examine whether hedonic (resp. nutritional) pairings and categorizations actually reflect hedonic (resp. nutritional) considerations [34]. Regarding the implicit pairing task, children were interviewed and their verbatims were collected to ensure that an association such as steak and chicken (coded as a nutritional association) was motivated by nutritional considerations (e.g., “they are both meat”), and that an association such as chicken and French fries (coded as a hedonic association) was motivated by hedonic considerations (e.g., “I like eating chicken with French fries”). The results of the qualitative analysis of children’s words led the authors to conclude that hedonic or nutritional pairings were congruently justified by hedonic or nutritional justifications, respectively, for all food triplets. Regarding the explicit categorization task, children were presented with different pictograms assumed to reflect the different categories (“yummy”, “yucky”, “makes you strong”, “makes you fat”, and “I don’t know this food”). Children were asked to describe each pictogram. Finally, the pictures that were correctly labeled for the purpose of the study by at least 95% of the children were selected. These categories labels were selected by the authors because they referred to concrete situations easily conceptualized by young children. They highlighted that the children preferentially chose concrete answers such as “makes you fat” or “makes you strong” instead of more abstract categories such as “good for your health” or “bad for your health”.

### Liking and healthiness perception

During face-to-face interviews, children rated their liking and health perception of the 10 buffet foods based on pictures of these foods. Children were presented with the 10 food pictures, one at a time, and rated their liking (i.e., “How much do you like this food?”) using a continuous scale ranging from “I do not like it at all” to “I like it very much”, which was coded from 0 to 10, respectively. After they rated their liking for all the foods, they were presented with the 10 food pictures again and rated their healthiness perceptions (i.e., “Is this food healthy?”) using a continuous scale ranging from “It is not healthy at all” to “It is very healthy”, coded from 0 to 10, respectively.

### Children’s anthropometrics

Weight (kg) was measured to the nearest 0.1 kg using a digital scale (Soehnle, Benfeld, Germany) while the children wore light clothes and no shoes; height (cm) was measured to the nearest 0.1 cm while the children stood without shoes using a stadiometer (Seca Leicester, Birmingham, UK). BMI was calculated and transformed into age- and sex-standardized z-scores (z-BMI) based on the French reference data [43].

### Hunger level

At the beginning of the session, the children indicated their hunger on a four-point scale ranging from (1) not hungry at all to (4) very hungry.

### Statistical analyses

To describe children’s perceptions of the buffet food assortment, liking and healthiness perception of healthy versus unhealthy foods were compared using Student’s T-tests. Multiple linear regression analyses were conducted to analyze the effect of the explicit and implicit hedonic scores as well as their interaction with regard to the (1) number of healthy food choices, (2) time spent to make the food choices, (3) difference between the liking ratings of the chosen and non-chosen foods (referred as ∆liking_*chosen-non_chosen*_), (4) difference between the healthiness ratings of the chosen and non-chosen foods (referred as ∆healthiness_*chosen-non_chosen*_), (5) difference between the liking ratings of the healthy and unhealthy foods (referred as ∆liking_*healthy-unhealthy*_), and (6) difference between the healthiness ratings of the healthy and unhealthy foods (referred as ∆healthiness_*healthy-unhealthy*_). We assumed that ∆liking_*chosen-non_chosen*_ (resp. ∆healthiness_*chosen-non_chosen*_) reflected the use of liking (resp. healthiness perception) as a criterion for food choice. All the participants completed the entire protocol, there was no missing data and the analyses were conducted on the total sample of 63 children. All of the multiple linear regression analyses presented in the results section included three continuous control variables: hunger level, age, and z-BMI. All statistical analyses were performed using SAS version 9.3 (SAS Institute, Inc., 2012 SAS® 9.3. Cary, NC). The level of significance was set at *P* = 0.05. The results are expressed as means ± SDs.

## Results

### Preliminary results

#### Perception of the food assortment

The liking and healthiness perception ratings of each buffet food are presented in Table 2. On average, the liking of healthy foods was similar to that of unhealthy foods (*M*_*liking_healthy*_ = 8.4 ± 1.4, *M*_*liking_unhealthy*_ = 7.9 ± 1.5; *t*(62) = 1.73, *P* = 0.09), whereas healthiness perception significantly differed between healthy and unhealthy foods (*M*_*healthiness_healthy*_ = 9.3 ± 0.8, *M*_*healthiness_unhealthy*_ = 3.7 ± 2.1; *t*(62) = 21.0, *P* < 0.0001). In addition, a Spearman’s correlation revealed that children’s health ratings were inversely linked with food ED (Spearman’s *ρ* = −0.68, *P* = 0.03). High ED is an indicator of poor nutritional quality in western countries [44, 45]. Thus, children’s perception of food healthiness matched an objective indicator of food nutritional quality. Moreover, no correlation was found between liking and healthiness for the 10 food items rated by the children (Spearman’s *ρ* = 0.12, *P* = 0.75).

**Table 2:** Liking and healthiness perception ratings of each buffet food (*n* = 63)

	Liking	Healthiness
Red apple	8.2 ± 2.2	9.6 ± 1.1
Banana	8.3 ± 2.4	9.1 ± 1.4
Kiwi	7.9 ± 3.4	9.3 ± 1.2
Applesauce	8.2 ± 2.8	9.4 ± 1.2
Strawberries	9.4 ± 1.6	9.1 ± 1.6
Donuts	7.1 ± 3.1	4.0 ± 3.3
Chocolate cake	8.0 ± 2.6	4.4 ± 3.2
Smarties®	8.2 ± 2.5	3.6 ± 2.7
Kinder Bueno®	8.4 ± 2.5	3.4 ± 2.6
Gold Bears®	8.1 ± 2.6	3.0 ± 2.9

Values are presented as the means ± SD

#### Description of children’s food choices

On average, the children chose 2.2 ± 1.1 healthy foods in 55.9 ± 24.2 s. All of the children ate all the foods that they chose. The liking ratings of the chosen foods were higher than those of the non-chosen foods (*M*_*liking_chosen*_ = 9.1 ± 0.87, *M*_*liking_non_chosen*_ = 7.3 ± 1.6; *t*(62) = 8.33, *P <* 0.0001), whereas healthiness perception did not significantly differ between the chosen and non-chosen foods (*M*_*healthiness_chosen*_ = 6.2 ± 1.9, *M*_*healthiness_non_chosen*_ = 6.8 ± 1.7; *t*(62) = −1.96, *P =* 0.055).

#### Description of children’s attitudes toward food

On average, children’s implicit attitudes toward food were primarily hedonic-based (*M*_*implicit_hedonic_score*_ = 75.7 ± 26.6%), as were their explicit attitudes, to a lesser extent (*M*_*explicit_hedonic_score*_ = 56.0 ± 23.4%). The implicit and explicit hedonic scores were not significantly correlated with each other (*r* = 0.09, *P* = 0.48).

### Primary results

#### Effect of explicit and implicit attitudes on children’s food choices

**Fig. 2:**
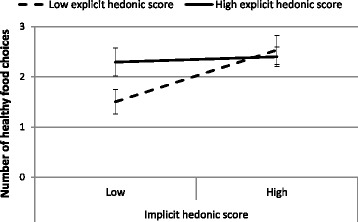
The effects of the relative dominance of nutrition- versus hedonic-based implicit and explicit attitudes on the number of healthy food choices. The four groups of children were created based on median splits of the implicit and explicit hedonic scores. Values are presented as the means ± SEM

The number of healthy buffet foods chosen significantly increased as a function of both the implicit hedonic score (*β* = 0.04, *95% CI* [0.011; 0.061], *P* = 0.01) and the explicit hedonic score (*β* = 0.05, *95% CI* [0.015; 0.088], *P* = 0.01). The interaction between the explicit and implicit hedonic scores was also significant (*β* = −0.001, *95% CI* [−0.001; −0.0001], *P* = 0.01). Median splits were performed with regard to the implicit and explicit hedonic scores to create four groups of children with contrasting attitudes toward food. As Fig. 2 shows, the children with both high implicit and explicit hedonic scores chose more healthy foods, as did the children with either high implicit or high explicit hedonic scores. Conversely, children with both low implicit and explicit hedonic scores chose fewer healthy buffet foods than those from the other groups.

#### Effect of explicit and implicit attitudes on the time spent to make food choices

The time that children took to choose was not related to their implicit hedonic scores (*β* = −0.23, *95% CI* [−0.85; 0.40], *P* = 0.47) or their explicit hedonic scores (*β* = −0.06, *95% CI* [−0.96; 0.85], *P* = 0.90).

#### Effect of explicit and implicit attitudes on the difference between the liking or healthiness ratings of the chosen and non-chosen foods

To further understand the relationship between the relative dominance of hedonic versus nutrition-based implicit and explicit attitudes toward food and children’s food choices, the effects of the implicit and explicit hedonic scores on ∆liking_*chosen-non-chosen*_ and ∆healthiness_*chosen-non-chosen*_ were tested. On average, ∆liking_*chosen-non-chosen*_ = 1.75 ± 1.67 and significantly differed from zero (*P* < 0.001), and ∆healthiness_*chosen-non-chosen*_ = −0.62 and did not significantly differ from zero (*P* = 0.055). The results of the linear regressions did not show an effect of the implicit (*β* = 0.02, *95% CI* [−0.03; 0.06], *P* = 0.50) and explicit (*β* = 0.03, *95% CI* [−0.04; 0.09], *P* = 0.38) hedonic scores on ∆liking_*chosen-non-*chosen_ but a significant effect of both implicit (*β* = 0.09, *95% CI* [0.03; 0.15], *P* = 0.004) and explicit (*β* = 0.13, *95% Cs* [0.04; 0.22], *P* = 0.01) hedonic scores as well as their interaction (*β* = −0.001, *95% CI* [−0.002; −0.0003], *P* = 0.01) on ∆healthiness_*chosen-non-chosen*_.

#### Effect of explicit and implicit attitudes on the difference between the liking or healthiness ratings of the healthy and unhealthy foods

We wondered whether the above results were because the children with low implicit and explicit hedonic scores liked healthy foods less than unhealthy foods; this effect might have led to a greater number of unhealthy food choices driven by liking. The effects of the implicit and explicit hedonic scores on ∆liking_*healthy-unhealthy*_ were tested. No significant effect of the implicit (*β* = 0.05, *95% CI* [−0.003; 0.10], *P* = 0.07) or explicit (*β* = 0.04, *95% CI* [−0.04; 0.11], *P* = 0.31) hedonic scores on ∆liking_*healthy-unhealthy*_ was found. In addition, we did not observe a significant effect of the implicit (*β* = −0.004, *95% CI* [−0.06; 0.05], *P* = 0.88) or explicit (*β* = −0.0003, *95% CI* [−0.08; 0.08], *P* = 0.85) hedonic scores on ∆healthiness_*healthy-unhealthy*_.

## Discussion

The present study explored the relationship between children’s implicit and explicit attitudes toward food. Specifically, we investigated the relative dominance of hedonic- versus nutrition-based attitudes, and children’s food choices with regard to a buffet offering highly appreciated healthy and unhealthy sweet foods. To assess children’s attitudes toward food, we used two tasks previously developed by Monnery-Patris et al. (2016) [34]. The implicit pairing task assessed the dominance of hedonic versus nutritional associations without criteria. This task likely assesses implicit attitudes toward food, which are defined as automatic and spontaneous [26]. In fact, implicit attitudes likely result from associative reasoning and reflect an individual’s level of exposure to a given association in one’s culture [46]. We assume that the implicit hedonic score from the implicit pairing task reflects passive learning of the relative dominance of hedonic versus nutritional considerations through successive food experiences. The explicit forced-choice categorization task assessed the dominance of hedonic versus nutritional categorizations when explicit classification criteria were given. This task likely assessed explicit attitudes toward food, which are defined as non-automatic and deliberative [26]. Because this task involved a direct analysis of the potential benefits of food consumption, either hedonic or nutritional, the explicit hedonic score may reflect what children are taught about food. To clearly establish a difference in the interpretation of the two tasks, we hypothesize that the implicit hedonic score reflects a passive learning and appropriation of cultural food values among children, whereas the explicit hedonic score reflects their intentional education of the hedonic and nutritional values of food through family expectations, media information about nutrition, and health promotion programs.

This study observed that both implicit and explicit attitudes significantly influenced children’s food choices. We observed that children with more hedonic-based implicit or explicit attitudes toward food chose healthier buffet foods. The influence of implicit and explicit hedonic scores on food choices was also moderated by a negative interaction indicating that children who had both low implicit and explicit hedonic scores were those who chose the fewest healthy food options. These results support Rozin et al.’s hypothesis that pleasure-oriented attitudes toward food are associated with healthier diets [38]. On one hand, we showed that the difference in liking between chosen and non-chosen foods was significantly positive, and neither implicit nor explicit hedonic scores affected this difference; in other words, children primarily chose the foods that they liked regardless of their attitudes. On the other hand, we showed that the difference in healthiness perception between the chosen and non-chosen foods was negative but not significantly different from zero; furthermore, this difference increased as a function of both implicit and explicit hedonic scores. Thus, when their implicit and explicit hedonic scores decreased, children chose the foods they perceived as less healthy, even though that they liked the healthy and unhealthy buffet foods equally.

Children with high hedonic scores chose healthier food options from a buffet offering similarly liked healthy and unhealthy foods. In this particular condition, having hedonic-based attitudes might have driven healthy food choices. In fact, hedonically oriented children chose the foods that they liked without considering the healthiness of foods, leading to a balanced choice of healthy and unhealthy food items. Conversely, children with both low implicit and explicit hedonic scores chose more unhealthy buffet food items and did not report a higher liking for unhealthy foods. Being unhealthy per se likely made unhealthy foods more attractive to them. At first sight, it could be surprising that the children most motivated to eat unhealthy foods were those with both implicit and explicit nutrition-based attitudes. Several hypotheses might explain why such an attitudinal pattern is associated with unhealthier food choices. Based on the interpretation of the tasks, having both implicit and explicit nutrition-based attitudes might reflect both a passive learning of and an intentional education about the nutritional value of foods in children [34]. This effect might be because of particular nutrition-focused familial contexts associated with restrictive parental feeding practices that enhanced children’s motivations to eat unhealthy foods during the experiment. For instance, children’s desire for a food increases significantly when its access is prohibited [47], and children consume more of a formerly forbidden food when they are finally allowed to eat it [48]. Children with both implicit and explicit nutrition-based attitudes might have a restricted access to unhealthy foods at home which consequently increased their selection and intake of these foods in the context of this experiment. In fact, the experimental session occurred in the absence of a parent and might have been perceived as particularly tempting because numerous highly appreciated sweets were available. Perceived restrictive parental feeding practices were not recorded in the present study. Additional research is needed to investigate the psychological and environmental factors associated with this nutrition-focused pattern of attitudes toward food in children. To avoid any potential counter-productive effect of this nutrition-focused attitudinal pattern, we suggest that shaping hedonic-based attitudes toward food by enhancing the attractiveness of healthy foods using pleasure-based strategies could be efficient to drive healthy food choices in children [40].

From a theoretical perspective, the present study was the first to investigate the ways that both implicit and explicit attitudes toward food predict children’s food choices. We found an interaction between implicit and explicit attitudes with regard to their influence on children’s buffet food choices, supporting the multiplicative predictive model [28]. Although the attitudes measures differed, this result is consistent with König et al. [31] who found a negative interaction between the effects of implicit and explicit attitudes on food choice using a multiple-option choice task involving the choice of a meal at a fake foods buffet. These authors specifically recorded the total amount of self-served sweets and fruit as well as studied their relationships with implicit and explicit attitudes toward these foods. König et al. highlighted a compensatory “one attitude is sufficient” effect because more sweets than fruit were chosen when at least one attitude (either implicit or explicit) showed a preference for sweets over fruit. Similarly, we found that when either the implicit or explicit hedonic score was high, children chose more healthy foods.

### Strengths and limitations

One strength of this study was its assessment of actual food choices among children in an ecologically valid eating situation. In fact, 80% of French 3- to 17-year-olds have a snack in the afternoon at least 4 times a week [49]; thus, snacking is a common practice. A limitation of the protocol is that the study occurred in a laboratory, which might have biased children’s food choices. Nevertheless, the laboratory context allowed us to accurately control certain environmental parameters. It ensured that each child made his or her own food choices under the same conditions so that these choices could be properly compared. Regarding food assortment, we chose highly liked healthy and unhealthy foods. Although both healthy and unhealthy foods were displayed in the buffet without packaging, certain unhealthy foods might have been recognized as branded products that may have influenced children’s food choices. Moreover, this particular assortment of food questions the generalizability of our findings. Notably, one may wonder whether implicit and explicit hedonic scores would have similarly predicted healthier food choices if the healthy food items had been less liked (e.g., if we had chosen vegetables instead of fruit). The answer might be negative. Knowing that liking was a strong predictor of children’s food choices independent of their implicit and explicit hedonic score, offering foods with contrasted liking could have driven all of the children to the same choice: they would have chosen the foods they liked (i.e., the unhealthy options). Thus, offering similarly liked healthy and unhealthy foods is strength of our experimental design because it enabled us to observe a fine tuning of food choices based on attitudes toward food. Moreover, the age range was quite large and it might have affected the results. The age of the participants was included as a control variable in all the statistical analyses (Additional file 1). However, the effect of age on children’s food choices was not significant. Thus, the wide age range strengthens the assumption that food choices were primarily influenced by children’s attitudes rather than by their age. The same is also true for z-BMI, also included as a control variable in all the statistical analyses (Additional file 1). Finally, in line with previous studies, we assumed that the implicit pairing task and the explicit categorization task measured the implicit and explicit relative dominance of hedonic- versus nutrition-based attitudes toward food, respectively [33, 34]. However, the implicit pairing task is likely to have captured something else than strictly speaking hedonic or nutritional aspects of food. In fact, pairing two meats together because “they are both meats” could reflect nutritional considerations but also usage (e.g., two meats are interchangeable within a meal, because they belong to the same food category). Conversely, pairing two foods together because they are good together could reflect hedonic considerations but also habits. However, there is still an opposition between cognitive associations (i.e., pairing two similar foods according to their taxonomic status) and affective associations (i.e., pairing two complementary foods reflects the anticipation of food consumption and of pleasure which may derive from it).

## Conclusion

Contrary to common belief, our experiment showed that having hedonic-based attitudes toward food predicted healthier food choices in children, whereas consistent implicit and explicit nutrition-based attitudes were associated with fewer healthy food choices from a buffet offering highly liked sweets of contrasting nutritional quality. These findings indicate that pleasure from eating could be an ally more than a threat regarding healthy eating in children, at least when liked healthy foods are available. These results are of particular interest from a public health perspective because they indicate that hedonic-based attitudes might be a lever to enhance healthy eating behaviors among children. It now appears important to develop further research to understand the etiology of children’s attitudes toward foods in order to provide insights on how to shape adequate children’s attitudes to guide them toward healthy food choices.

## Additional file

Additional file 1: Complete description of the multiple linear regression analyses*.* The Additional file 1 presents the results of the 5 multiple linear regression analyses including the DF, F-value, P > F, β, 95% CI and P > |t| for the three variables of interest, namely the implicit score, the explicit score and the interaction between the scores, and for the three control variables, namely age, z-BMI and hunger level. (DOCX 26 kb)

## Abbreviations

z-BMI: Body mass index z-score
ED: Energy density
