# Supplementary material for: Do hedonic- versus nutrition-based attitudes toward food predict food choices? a cross-sectional study of 6- to 11-year-olds
Source: Int J Behav Nutr Phys Act. 2017 Nov 25;14:162. doi: 10.1186/s12966-017-0618-4 (PMC5702150; doi:10.1186/s12966-017-0618-4)
Supplement: Additional file 1: — Complete description of the multiple linear regression analyses. The Additional file 1 presents the results of the 5 multiple linear regression analyses including the DF, F-value, P > F, β, 95% CI and P > |t| for the three variables of interest, namely the implicit score, the explicit score and the interaction between the scores, and for the three control variables, namely age, z-BMI and hunger level. (DOCX 26 kb) [file 12966_2017_618_MOESM1_ESM.docx]

|  | Number of healthy food choices (*R^2^* = 0.27) | | | | | | | Time spent to make the food choices (*R^2^* = 0.10) | | | | | | |
| --- | --- | --- | --- | --- | --- | --- | --- | --- | --- | --- | --- | --- | --- | --- |
| Predictors | *DF (effect, error)* | *F-value* | *P > F* | *β* | *95% CI* | | *P > \|t\|* | *DF (effect, error)* | *F-value* | *P > F* | *β* | *CI* | | *P > \|t\|* |
| Implicit score | (1,56) | 8.25 | 0.006 | 0.04 | 0.01 | 0.06 | 0.01 | (1,56) | 0.54 | 0.47 | -0.23 | -0.85 | 0.40 | 0.47 |
| Explicit score | (1,56) | 8.02 | 0.009 | 0.05 | 0.02 | 0.09 | 0.01 | (1,56) | 0.01 | 0.90 | -0.06 | -0.96 | 0.85 | 0.90 |
| Implicit × explicit score | (1,56) | 6.36 | 0.01 | -0.001 | -0.001 | -0.0001 | 0.01 | (1,56) | 0.04 | 0.83 | 0.001 | -0.01 | 0.01 | 0.83 |
| Age | (1,56) | 1.89 | 0.17 | -0.11 | -0.28 | 0.05 | 0.17 | (1,56) | 0.37 | 0.55 | 1.25 | -2.88 | 5.39 | 0.55 |
| z-BMI | (1,56) | 2.25 | 0.14 | 0.10 | -0.03 | 0.23 | 0.14 | (1,56) | 0.31 | 0.58 | 0.94 | -2.48 | 4.37 | 0.58 |
| Hunger level | (1,56) | 5.04 | 0.03 | -0.36 | -0.67 | -0.04 | 0.03 | (1,56) | 3.15 | 0.08 | -7.11 | -15.15 | 0.92 | 0.08 |
|  | ∆liking*_chosen-non_chosen_* (*R^2^* = 0.03) | | | | | | | ∆healthiness*_chosen-non_chosen_ (R^2^ = 0.22)* | | | | | | |
| Predictors | *DF (effect, error)* | *F-value* | *P > F* | *β* | *CI* | | *P > \|t\|* | *DF (effect, error)* | *F-value* | *P > F* | *β* | *CI* | | *P > \|t\|* |
| Implicit score | (1,56) | 0.45 | 0.50 | 0.02 | -0.03 | 0.06 | 0.50 | (1,56) | 8.84 | 0.004 | 0.09 | 0.03 | 0.15 | 0.004 |
| Explicit score | (1,56) | 0.78 | 0.38 | 0.03 | -0.04 | 0.09 | 0.38 | (1,56) | 8.38 | 0.01 | 0.13 | 0.04 | 0.22 | 0.01 |
| Implicit × explicit score | (1,56) | 0.59 | 0.44 | 0.00 | 0.00 | 0.00 | 0.44 | (1,56) | 6.39 | 0.01 | -0.001 | -0.002 | -0.0003 | 0.01 |
| Age | (1,56) | 0.00 | 0.99 | 0.00 | -0.30 | 0.29 | 0.99 | (1,56) | 0.90 | 0.35 | -0.19 | -0.59 | 0.21 | 0.35 |
| z-BMI | (1,56) | 0.17 | 0.68 | -0.05 | -0.29 | 0.19 | 0.68 | (1,56) | 2.13 | 0.15 | 0.24 | -0.09 | 0.56 | 0.15 |
| Hunger level | (1,56) | 0.15 | 0.70 | 0.11 | -0.46 | 0.68 | 0.70 | (1,56) | 0.24 | 0.62 | -0.19 | -0.97 | 0.58 | 0.62 |
|  | ∆liking*_healthy-unhealthy_ (R^2^ = 0.22)* | | | | | | | ∆healthiness*_healthy-unhealthy_ (R^2^ = 0.11)* | | | | | | |
| Predictors | *DF (effect, error)* | *F-value* | *P > F* | *β* | *CI* | | *P > \|t\|* | *DF (effect, error)* | *F-value* | *P > F* | *β* | *CI* | | *P > \|t\|* |
| Implicit score | (1,56) | 3.51 | 0.07 | 0.05 | -0.003 | 0.10 | 0.07 | (1,56) | 0.02 | 0.88 | -0.004 | -0.06 | 0.05 | 0.88 |
| Explicit score | (1,56) | 1.06 | 0.31 | 0.04 | -0.04 | 0.11 | 0.31 | (1,56) | 0.00 | 0.99 | -0.0003 | -0.08 | 0.08 | 0.99 |
| Implicit × explicit score | (1,56) | 2.08 | 0.16 | 0.00 | 0.00 | 0.00 | 0.16 | (1,56) | 0.03 | 0.85 | -0.0001 | -0.001 | 0.001 | 0.85 |
| Age | (1,56) | 0.22 | 0.64 | -0.08 | -0.41 | 0.26 | 0.64 | (1,56) | 4.54 | 0.04 | 0.39 | 0.02 | 0.75 | 0.04 |
| z-BMI | (1,56) | 8.48 | 0.01 | 0.39 | 0.12 | 0.67 | 0.01 | (1,56) | 0.07 | 0.79 | -0.04 | -0.33 | 0.25 | 0.79 |
| Hunger level | (1,56) | 0.12 | 0.73 | -0.11 | -0.76 | 0.54 | 0.73 | (1,56) | 0.08 | 0.78 | -0.10 | -0.80 | 0.60 | 0.78 |
